# Association of statin therapy with clinical outcomes in patients with vasospastic angina: Data from Korean health insurance review and assessment service

**DOI:** 10.1371/journal.pone.0210498

**Published:** 2019-01-30

**Authors:** So Jin Park, Hyejeong Park, Danbee Kang, Taek Kyu Park, Jinkyeong Park, Joongbum Cho, Chi Ryang Chung, Kyeongman Jeon, Eliseo Guallar, Juhee Cho, Gee Young Suh, Jeong Hoon Yang

**Affiliations:** 1 Department of Pharmaceutical Services, Samsung Medical Center, Seoul, Republic of Korea; 2 Center for Clinical Epidemiology, Samsung Medical Center, Seoul, Republic of Korea; 3 Division of Cardiology, Department of Medicine, Samsung Medical Center, Sungkyunkwan University School of Medicine, Seoul, Republic of Korea; 4 Department of Critical Care Medicine, Samsung Medical Center, Sungkyunkwan University School of Medicine, Seoul, Republic of Korea; 5 Department of Clinical Research Design & Evaluation, SAIHST, Sungkyunkwan University, Seoul, Republic of Korea; 6 Departments of Epidemiology and Medicine, and Welch Center for Prevention, Epidemiology, and Clinical Research, Johns Hopkins Bloomberg School of Public Health, Baltimore, Maryland, United States of America; University of Bologna, ITALY

## Abstract

There is conflicting evidence for the clinical benefit of statin therapy in patients with vasospastic angina (VSA). We investigated the association of statin therapy with clinical outcomes in relatively large populations with clinically suspected VSA from a nationwide population-based database. Data were collected from the Health Insurance Review and Assessment database records of 4,099 patients that were in an intensive care unit with VSA between January 1, 2008 and May 31, 2015. We divided the patients into a statin group (n = 1,795) and a non-statin group (n = 2,304). The primary outcome was a composite of cardiac arrest and acute myocardial infarction (AMI). The median follow-up duration was 3.8 years (interquartile range: 2.2 to 5.8 years). Cardiac arrest or AMI occurred in 120 patients (5.2%) in the statin group, and 97 patients (5.4%) in the non-statin group (*P* = 0.976). With inverse probability of treatment weighting, there was no significant difference in the rate of cardiac arrest or AMI between the two groups (adjusted hazard ratio [HR], 0.99; 95% confidence interval [CI], 0.76–1.30; *P* = 0.937), or even between the non-statin group and high-intensity statin group (adjusted HR, 1.08; 95% CI, 0.69–1.70; *P* = 0.75). The beneficial association of statin use with the primary outcome was consistently lacking across the various comorbidity types. Statin therapy was not associated with reduced cardiac arrest or AMI in patients with VSA, regardless of statin intensity. Prospective, randomized trials will be needed to confirm our findings.

## Introduction

Although the precise mechanism of coronary artery spasm has not been fully established, several factors such as endothelial dysfunction, smooth muscle hyperreactivity, autonomic dysfunction, abnormal coronary microvascular function, and vascular inflammation can also influence vasospasm [1–6]. Statin (3-hydroxy-3-methyl-glutaryl-coenzyme A reductase inhibitor) therapy became a mainstay for the medical treatment and prevention of atherosclerotic cardiovascular disease (ASCVD). Previous studies have shown that statin is associated with improvement in endothelial dysfunction, increases in nitric oxide bioavailability, inhibition of inflammatory responses, and stabilization of atherosclerotic plaques [5,7]. Theoretically, the pleomorphic effects of statin may provide a cardiovascular benefit beyond that expected from low density lipoprotein-cholesterol lowering alone in the setting of vasospastic angina (VSA). However, two recently published studies [8,9] showed no association of statin therapy with reduced cardiac death and recurrent myocardial infarction in VSA without significant stenosis, even though statin therapy was associated with reductions in mortality and future ASCVD risk in previous randomized trials with various ASCVD populations [10,11]. These studies of VSA had two major limitations: they had a limited population, and the association between high-intensity statin and clinical outcomes was not seen because most of the study patients were treated with low- to moderate-intensity statin. Therefore, we investigated the association of statin therapy with clinical outcomes and whether the clinical impacts of non–high-dose statin and high-dose statin are different, in relatively large populations with VSA from a nationwide population-based database.

## Materials and methods

### Study population

We conducted a retrospective cohort analysis of the Health Insurance Review and Assessment (HIRA) database from the Korean Ministry of Health and welfare. HIRA provides both sample and customized datasets when the applicant submits necessary information and forms [12]. In this study, we used customized dataset and it included all admissions to the intensive care unit in Korea during the study period. The study population consisted of all patients >18 years of age admitted to an intensive care unit with VSA from January 1, 2008 to May 31, 2015. We defined these admissions using the HIRA Service codes for cost claims for intensive care unit stays (AJ100-AJ590900). These codes are based on those of the Korean Classification of Diseases, 6^th^ Edition, which is the modified version of the International Classification of Diseases, 10^th^ Revision (ICD-10) adapted for use in the Korean health system [13]. All intensive care unit stays during the same hospitalization were considered as a single admission to the intensive care unit. Similarly, hospital stays separated by <2 days were considered as the same hospital admission. Among them, patients with VSA (n = 8,999) were defined using a combination of ICD-10 codes for VSA (I201) and Korean National Health Insurance (KNHI) codes for coronary angiography procedures (HA670, HA680, HA681, HA682). Then, we excluded patients who were admitted with VSA (ICD-10 code I201) up to six months before the index hospitalization, who were admitted with acute myocardial infarction (AMI) (ICD-10 codes I21, I22, I23) up to six months before the index hospitalization and within the admission (n = 1,733), or who had taken a statin up to a year before the index hospitalization (n = 2,347). We also excluded patients who had undergone percutaneous coronary intervention (KNHI codes M6551-M6552, M6561-M6564, M6571-M6572) or bypass surgery (KNHI codes O1641-O1642, OA641-OA642) between six months before the admission and the index hospitalization and admission (n = 717), who died during the index hospitalization (n = 68), or who had no outpatient claims after discharge (n = 35). The study was reviewed by the Institutional Review Board of Samsung Medical Center (protocol 2015-11-17), and was exempted because we only accessed de-identified, previously collected administrative data.

### Measurements

To define statin use and other information on the underlying disease and comorbidities, procedures, prescriptions, and demographic characteristics, we used ICD-10 codes [14] and Korean drug and Anatomical Therapeutic Chemical Classification codes. Statins were identified using the following generic names: atorvastatin, fluvastatin, lovastatin, pitavastatin, pravastatin, rosuvastatin, and simvastatin, from admission to first outpatient care after discharge. In addition, we classified patients with VSA into two groups: non-high intensity statin (atorvastatin [10 mg, 20 mg], fluvastatin [20 mg, 40 mg, 80 mg], lovastatin [20 mg], pitavastatin [1 mg, 2 mg, 4 mg], pravastatin [5 mg, 10 mg, 20 mg, 40 mg], rosuvastatin [5 mg, 10 mg], or simvastatin [5 mg, 10 mg, 20 mg, 40 mg, 80 mg]) and high-intensity statin groups (atorvastatin [40 mg, 80 mg] or rosuvastatin [20 mg]). We also identified use of medications related to variant angina as aspirin, calcium-channel blocker (amlodipine, barnidipine, benidipine, cilnidipine, efonidipine, felodipine, isradipine, lacidipine, lercanidipine, manidipine, nicardipine, nifedipine, nilvadipine, nimodipine, nisoldipine, and nitrendipine), nitrate (isosorbide dinitrate, isosorbide mononitrate, nitroglycerin, and nitroprusside), nicorandil, trimetazidine, angiotensin-converting enzyme inhibitor (ACE inhibitor) (alacepril, benazepril, captopril, cilazapril, enalapril, imidapril, lisinopril, moexipril, perindopril, quinapril, ramipril, temocapril, and zofenopril), and angiotensin receptor blocker (candesartan, eprosartan, fimasartan, irbesartan, losartan, olmesartan, telmisartan, and valsartan). Comorbidities were summarized using the Charlson Comorbidity Index [15,16]. We also included hypertension (I10-I15). Procedures of interest included continuous renal replacement therapy (O7051-7054) and extracorporeal membrane oxygenation (O1901-O1904, material codes; CAPIOX EBS CIRCUIT [G5401008], QUADROX PLS [G5501050], and CAPIOX EBS PMP CIRCUIT [G5501008]).

### Study outcomes

The primary outcome was a composite of cardiac arrest and AMI after discharge. To define post-admission outcomes, we linked the personal identification number of each study participant to the 2008–2016 inpatient claim data from the admission result database. Cardiac arrest was defined using the claim codes for cardiopulmonary resuscitation (HIRA or KNHI codes M5871, M5873, M5874, M5875, M5876, and M5877), and AMI was defined using the ICD-10 codes I21, I22, and I23 after discharge.

### Statistical analysis

Using the model, we estimated hazard ratios (HR) with 95% confidence intervals (CI) for the cumulative incidence of the composite of cardiac arrest and AMI associated with the presence of arrest, adjusted for other risk factors. We adjusted for age, sex, hypertension, diabetes mellitus, admission type, spasm provocation test, year of admission, aspirin, calcium-channel blocker, nitrate, nicorandil, trimetazidine, ACE inhibitor, and angiotensin receptor blocker. Since patients admitted to the same hospital could be correlated, we used a multivariable mixed-effects Cox regression analysis. The model was performed with hospital of admission as a random intercept to account for clustering by hospital. In addition, since we compared participants with and without statin treatment, we also used inverse probability of treatment weighting (IPTW) to correct for potential selection bias in this group. IPTW was obtained from a logistic regression model including age, sex, hypertension, diabetes mellitus, admission type, spasm provocation test, year, aspirin, calcium-channel blocker, nitrate, nicorandil, trimetazidine, ACE inhibitor, and angiotensin receptor blocker. All analyses reported were corrected for IPTW [17]. We considered a *P*-value of <0.05 as significant for all analyses. Statistical analysis was performed using SAS Visual Analytics.

## Results

### Baseline characteristics

**Fig 1:**
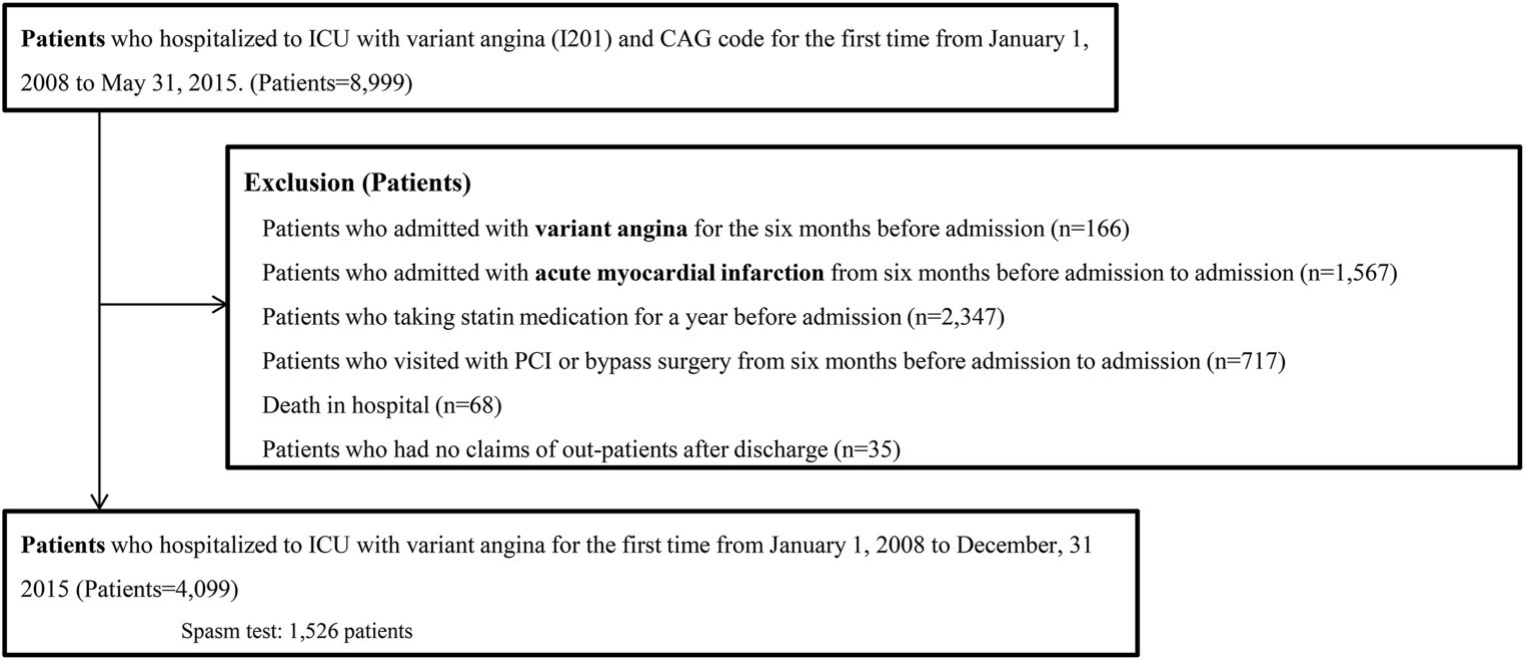
Study design and population. ICU: intensive care unit, PCI: percutaneous coronary intervention, CAG: coronary angiography.

A total of 4,099 patients with VSA were analyzed (Fig 1). Among them, patients underwent spasm testing were 1,526 (37.2%) and statins were prescribed for 2,304 (56.2%). Within that group, 460 patients were treated with high-intensity statin therapy. Baseline patient characteristics are listed in Table 1. Compared to patients not treated with statin, those treated with statin were older and included more men; had a higher incidence of hypertension or diabetes mellitus; had a higher admission rate from the emergency room; and were more likely to take aspirin, a calcium-channel blocker, nitrate, nicorandil, trimetazidine, an ACE inhibitor, or an angiotensin receptor blocker.

**Table 1:** Baseline characteristics of the clinically suspected vasospastic angina patients.

	Overall	Without statin	With statin	
	(N = 4,099)	(N = 1,795)	(N = 2,304)	*P* value
Age, years	54.2 ± 12.1	53.4 ± 12.5	54.9 ± 11.6	<0.001
Age ≥ 65 yrs	820 (20.0)	337 (18.8)	483 (21.0)	0.082
Male	2,950 (72.0)	1,244 (69.3)	1,706 (74.1)	<0.001
Hypertension	2,134 (52.1)	869 (48.4)	1,265 (54.9)	<0.001
Diabetes mellitus	1,496 (36.5)	617 (34.4)	879 (38.2)	0.013
Chronic kidney disease	40 (1.0)	23 (1.3)	17 (0.7)	0.079
Charlson index	1.1 ± 1.7	1.1 ± 1.7	1.1 ± 1.6	0.913
Tertiary referrer hospital	2,117 (51.7)	927 (51.6)	1,190 (51.7)	0.997
Admission from emergency room	3,170 (77.3)	1,348 (75.1)	1,822 (79.1)	0.003
Spasm provocation test	1,526 (37.2)	742 (41.3)	784 (34.0)	<0.001
Year				<0.001
July 2007–2009	961 (23.4)	479 (26.7)	482 (20.9)	
2010–2012	1,683 (41.1)	779 (43.4)	904 (39.2)	
2013–2015	1,455 (35.5)	537 (29.9)	918 (39.8)	
Medication at discharge				
Aspirin	3,705 (90.4)	1,528 (85.1)	2,177 (94.5)	<0.001
Calcium-channel blocker	3,449 (84.1)	1,476 (82.2)	1,973 (85.6)	0.003
Nitrate	3,619 (88.3)	1,547 (86.2)	2,072 (89.9)	<0.001
Nicorandil	1,848 (45.1)	740 (41.2)	1,108 (48.1)	<0.001
Trimetazidine	714 (17.4)	265 (14.8)	449 (19.5)	<0.001
ACE inhibitor	777 (19.0)	242 (13.5)	535 (23.2)	<0.001
Angiotensin receptor blocker	592 (14.4)	232 (12.9)	360 (15.6)	0.015
Extracorporeal membrane oxygenation	13 (0.3)	7 (0.4)	6 (0.3)	0.464
Continuous renal replacement therapy	14 (0.3)	9 (0.5)	5 (0.2)	0.121

Values are number of patients (%) or mean ± standard deviation.

ACE inhibitor, Angiotensin-converting enzyme inhibitors

### Clinical outcomes

**Fig 2:**
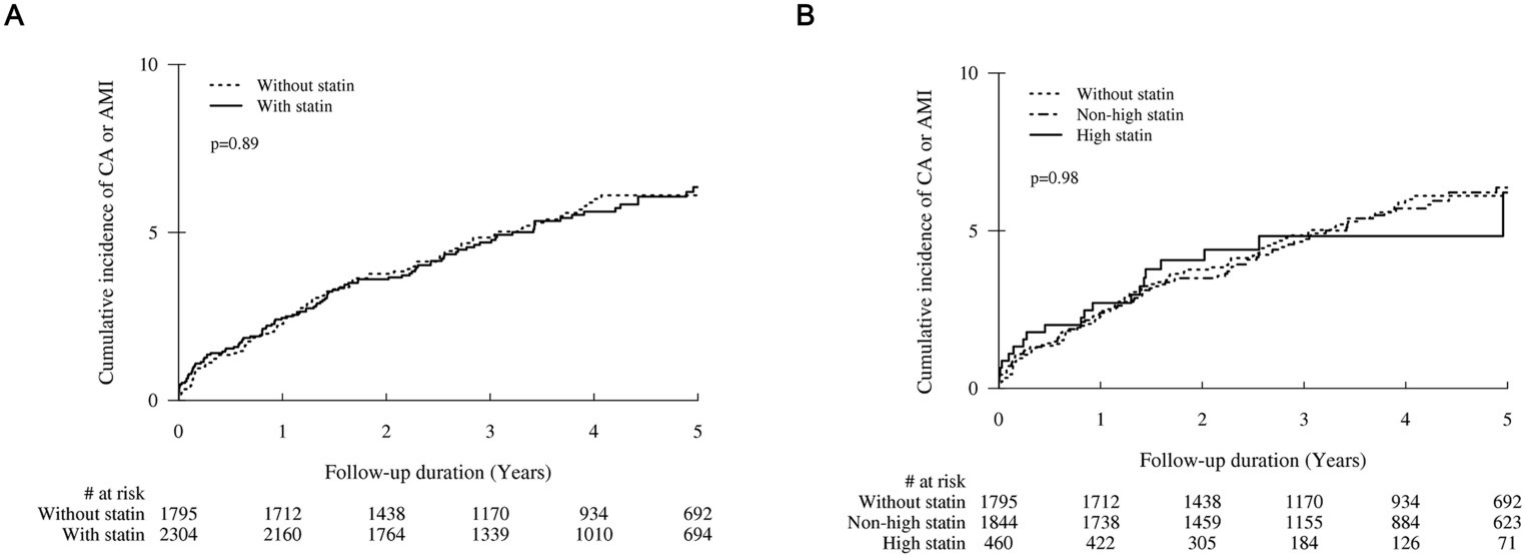
Kaplan-Meier curves of the composite of cardiac arrest and acute myocardial infarction. (A) Statin versus non-statin (B) Among non-statin, non–high-dose statin, and high-dose statin.

**Fig 3:**
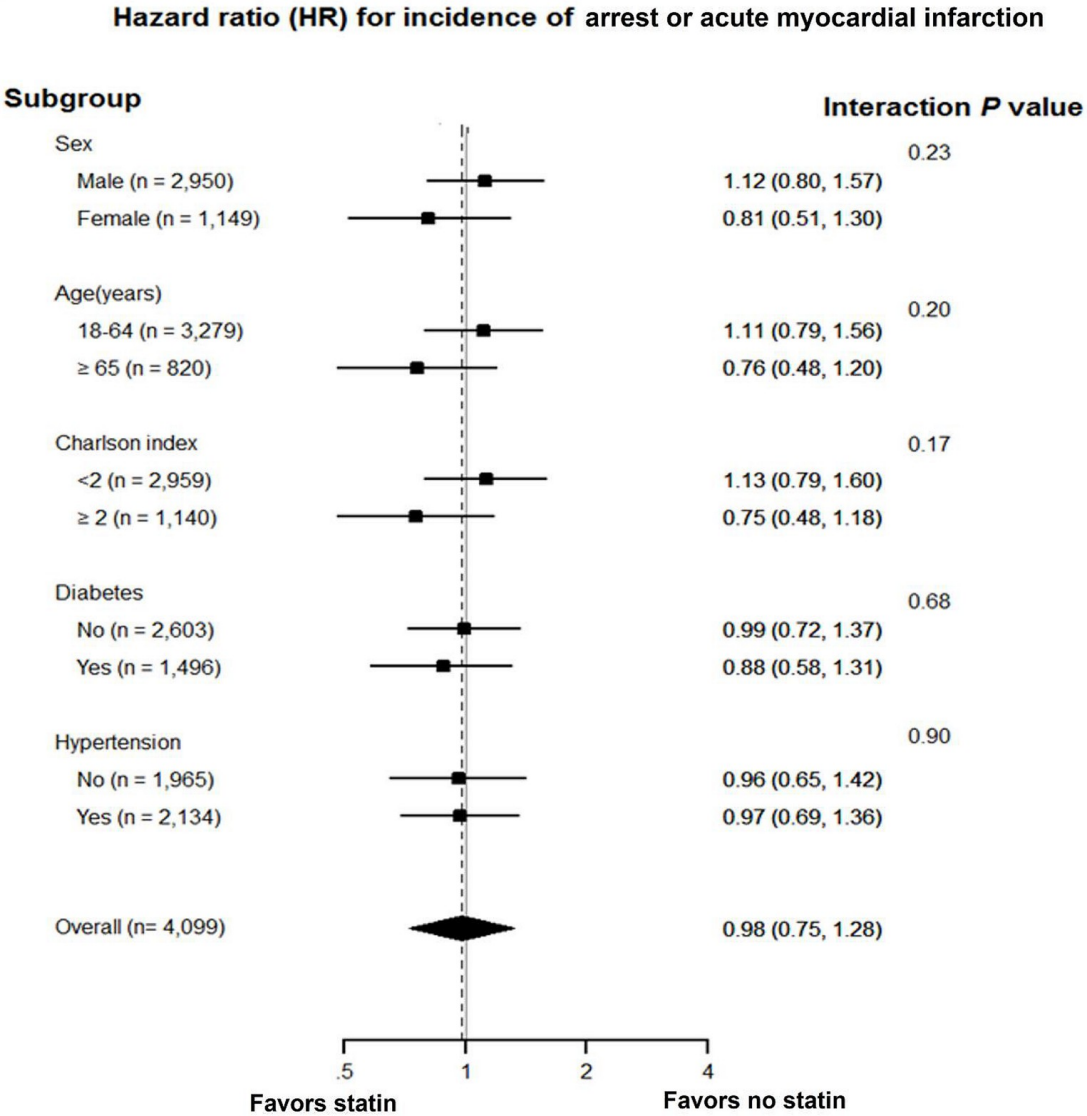
Subgroup analysis. Hazard ratios for the composite of cardiac arrest and acute myocardial infarction were estimated using the inverse probability of treatment weighting method among various subgroups.

Table 2 lists the clinical outcomes. The median follow-up duration was 3.8 years (interquartile range: 2.2 to 5.8 years). During follow-up after discharge, cardiac arrest or AMI occurred in 217 patients. The cumulative incidence of cardiac arrest or AMI was similar in patients treated with or without statin over the entire follow-up period (*P* = 0.98; Fig 2A). On multivariable analysis, the two groups had similar risks of the composite of cardiac arrest and AMI (adjusted HR, 0.98; 95% CI, 0.75–1.28; *P* = 0.899; Table 2), cardiac arrest (adjusted HR, 0.79; 95% CI, 0.50–1.26; *P* = 0.326; Table 2), and acute myocardial infarction (adjusted HR, 1.20; 95% CI, 0.88–1.62; *P* = 0.24; Table 2). Similarly, there was no significant difference in the rate of cardiac arrest or AMI between the two groups (adjusted HR, 0.99; 95% CI, 0.76–1.30; *P* = 0.937; Table 2) on IPTW. To identify whether the clinical impacts of non–high-dose statin and high-dose statin were different, we classified the patients with statin into non–high-intensity and high-intensity statin groups. On multivariable analysis, there was no significant difference even between the non-statin group and the high-intensity statin group (adjusted HR, 1.08; 95% CI, 0.69–1.70; *P* = 0.75; Fig 2B and Table 3) as well as between the non-statin group and the non–high-intensity statin group (adjusted HR, 0.98; 95% CI, 0.74–1.30; *P* = 0.891; Fig 2B and Table 3). Additionally, a lack of benefit of statin therapy with respect to the primary outcome was consistent across various subgroups (Fig 3) and there were no significant interactions between use of statin and the composite of cardiac arrest and AMI among all the subgroups (all *P*-values for interaction >0.10).

**Table 2:** Clinical outcomes in the clinically suspected vasospastic angina patients.

	Without statin	With statin	Univariable		Multivariable*		IPTW†	
	(N = 1,795)	(N = 2,304)	HR (95% CI)	*P* value	HR (95% CI)	*P* value	HR (95% CI)	*P* value
Cardiac arrest or Myocardial infarction	97 (5.4)	120 (5.2)	1.02 (0.78–1.33)	0.888	0.98 (0.75–1.28)	0.899	0.99 (0.76–1.30)	0.937
Cardiac arrest	46 (2.6)	46 (2.0)	0.83 (0.54–1.29)	0.404	0.79 (0.50–1.26)	0.326	0.80 (0.50–1.28)	0.351
Myocardial infarction	61 (3.4)	90 (3.9)	1.21 (0.91–1.61)	0.192	1.20 (0.88–1.62)	0.245	1.19 (0.88–1.62)	0.267

CI, confidence interval; HR, hazard ratio; IPTW, inverse probability-of-treatment weight.

Values are n (%).

*Adjusted for age, gender, hypertension, diabetes mellitus, admission type, spasm provocation test, year, aspirin, calcium-channel blocker, nitrate, nicorandil, trimetazidine, ACE inhibitor and angiotensin receptor blocker.

^†^Obtained from a logistic regression model included age, gender, year, aspirin, calcium-channel blocker, nitrate, nicorandil, trimetazidine, ACE inhibitor and angiotensin receptor blocker.

**Table 3:** Clinical outcomes in the clinically suspected vasospastic angina patients according to intensity of statin.

	Without statin (N = 1,795)	Non-high intensity statin (N = 1,844)		High intensity statin (N = 460)	
	HR (95% CI)	HR (95% CI)	*P* value	HR (95% CI)	*P* value
Cardiac arrest or Myocardial infarction					
Number of case (%)	97 (5.4)	100 (5.4)		20 (4.4)	
Unadjusted HR (95% CI)	Reference	1.03 (0.78–1.35)	0.859	0.99 (0.63–1.56)	0.965
Adjusted HR (95% CI)*^,^^†^	Reference	0.98 (0.74–1.30)	0.891	1.08 (0.69–1.70)	0.750
Adjusted HR (95% CI)*^,^^‡^	Reference	0.98 (0.73–1.31)	0.898	1.15 (0.72–1.83)	0.561
Cardiac arrest					
Number of case (%)	46 (2.6)	35 (1.9)		11 (2.4)	
Unadjusted HR (95% CI)	Reference	0.76 (0.47–1.23)	0.265	1.18 (0.63–2.18)	0.610
Adjusted HR (95% CI)*^,^^†^	Reference	0.73 (0.44–1.21)	0.217	1.26 (0.67–2.38)	0.478
Adjusted HR (95% CI)*^,^^‡^	Reference	0.73 (0.43–1.23)	0.233	1.41 (0.75–2.64)	0.282
Myocardial infarction					
Number of case (%)	61 (3.4)	79 (4.3)		11 (2.4)	
Unadjusted HR (95% CI)	Reference	1.28 (0.95–1.74)	0.108	0.86 (0.45–1.62)	0.631
Adjusted HR (95% CI)*^,^^†^	Reference	1.25 (0.90–1.73)	0.187	0.98 (0.51–1.87)	0.941
Adjusted HR (95% CI)*^,^^‡^	Reference	1.23 (0.89–1.70)	0.213	0.98 (0.49–1.98)	0.958

CI, confidence interval; HR, hazard ratio.

Values are n (%).

*Adjusted for age, gender, hypertension, diabetes mellitus, admission type, spasm provocation test, year, aspirin, calcium-channel blocker, nitrate, nicorandil, trimetazidine, ACE inhibitor and angiotensin receptor blocker.

^†^Using multilevel logistic regression.

^‡^Using inverse probability of treatment weighting with multilevel logistic regression. And obtained from a logistic regression model included age, gender, year, aspirin, calcium-channel blocker, nitrate, nicorandil, trimetazidine, ACE inhibitor and angiotensin receptor blocker.

## Discussion

In this study, we investigated the association between statin therapy and clinical outcomes, and assessed whether clinical impacts of statin intensity are different in patients with VSA selected from a nationwide population-based database. Our results indicate that statin therapy was not associated with reduced cardiac arrest or AMI in patients with clinically suspected VSA. High-dose statin was not associated with improved clinical outcomes, either. Furthermore, a lack of benefit of statin therapy on cardiac arrest or AMI was constantly observed across various subgroups.

The pleiotropic effects of statin on endothelial cell dysfunction and vascular inflammation have been expected to improve clinical outcomes of VSA, because they are considered to be related to VSA pathophysiology [7,18]. Actually, Kim et al demonstrated that atorvastatin was associated with improvement in endothelial cell dysfunction even over a short follow-up duration in patients with VSA [19]. In real-world practice, the results of adding a statin to vasodilator-based therapy could infer the following: improvement of lipid metabolism, especially an increase of high-density lipoprotein cholesterol and a reduction of malondialdehyde-modified low-density lipoprotein, would reduce vascular contractility by inhibiting atherosclerotic progress [20,21]. However, two recently published studies in patients with VSA but without significant coronary artery stenosis showed no association between statin therapy and cardiac death or recurrent myocardial infarction. Oh et al concluded that statin therapy could not improve long-term clinical outcomes, even readmission due to recurrent chest pain, in patients with VSA and without significant coronary artery disease [8]. Although Ishii et al showed that statin therapy was a significant predictor for reduction of major adverse cardiac events, the significance was from reduction of recurrent chest pain due to vasospasm, and there were no significant differences in hard endpoints such as cardiac death and acute myocardial infarction between the statin and non-statin groups [9]. At the same time, these two studies were single-center studies and included relatively small numbers of patients; thus, they did not analyze whether the prognosis differs in patients with VSA according to intensity of statin therapy. Conversely, the present study included a large sample size from a nationwide population using HIRA data, which enabled a subgroup analysis of statin intensity; and the study reflected current real-world practice such as high rates of use of statins, renin-angiotensin system blockers, or various vasodilators. In addition, we tried to include only patients with VSA without significant coronary artery stenosis; we did so by excluding patients who underwent percutaneous coronary intervention or coronary artery bypass graft during the index hospitalization as well as those with a history of coronary artery revascularization. Consistent with the findings of the previous two studies, this study also showed that there was no significant difference with respect to the hard endpoint between the statin group and the non-statin group. A large-scale, prospective randomized trial will be required to confirm these results.

High-intensity statin therapy has shown a superior survival advantage and reduction in disease progression over low- or moderate-intensity statin therapy in studies of various ASCVD populations [22–25]. Based on this evidence, the 2013 American College of Cardiology/American Heart Association guidelines recommend the use of high-intensity statin therapy among all patients 75 years or younger with ASCVD [26]. Until now, there have been no data on the impact of high-intensity statin therapy on clinical outcomes in patients with VSA. In this study, however, the use of high-intensity statin therapy was not associated with a reduction of readmission due to cardiac arrest or AMI. Although the reason for these findings is unclear, there are several possible explanations. First, the use of high dose statin might not have additional benefit to improve the prognosis of VSA. A previous study showed that the degree of flow-mediated vasodilation change at 6 months did not differ between atorvastatin 10 mg treatment and 40 mg treatment groups, with the caveat that the study had a small sample size [19]. Second, patients receiving high-intensity statin might be overall high-risk patients. The lack of additional benefit of high-intensity statin in our study may result from differences in baseline characteristics. While we addressed this by performing IPTW to adjust for those differences between the groups, we were not able to correct for unmeasured variables because of inherent limitations of HIRA data. Third, the 3.8-year median follow-up may have been insufficient to evaluate the benefit of statins in patients with VSA. The meta-analysis data from the Cholesterol Treatment Trialists’ Collaboration in 2010 showed that the benefits of statin were significant within the first year, but were greater in subsequent years [27,28].

This study has several limitations. First, it was an observational, retrospective study, which may have had selection bias and confounding factors. To minimize bias, we excluded patients who were prescribed statins a year before the index period of interest, verified the results in an IPTW-adjusted population, and used a multivariable mixed-effects Cox regression analysis to reduce confounding effects. Second, this study has an inherent limitation regarding the definition of the vasospastic angina as it is based on hospital coding rather than diagnostic criteria for vasospastic angina. We could not identify angiographic extent of coronary artery disease because our registry did not contain the detailed angiographic information. Third, although medication adherence is important, we could only determine if the prescription was issued and not if the patient actually took the medication. Lastly, because we only studied patients admitted to the intensive care unit, the results might not be generalizable to mild VSA with outpatient follow-up.

## Conclusions

Statin therapy was not associated with reductions in cardiac arrest or AMI regardless of statin intensity. Furthermore, a lack of benefit of statin therapy on cardiac arrest or AMI was observed across various subgroups.

## Supporting information

S1 Table Clinical outcomes in the angiographically suspected vasospastic angina patients with or without statin. (DOCX)

## Abbreviations

ACE inhibitor: Angiotensin-converting enzyme inhibitor
ASCVD: Atherosclerotic cardiovascular disease
CI: Confidence interval
HIRA: Health Insurance Review and Assessment
HR: Hazard ratio
ICD-10: International Classification of Diseases, 10^th^ Revision
IPTW: Inverse probability of treatment weighting
KNHI: Korean National Health Insurance
VSA: Vasospastic angina
