# Supplementary material for: Association of statin therapy with clinical outcomes in patients with vasospastic angina: Data from Korean health insurance review and assessment service
Source: PLoS One. 2019 Jan 30;14(1):e0210498. doi: 10.1371/journal.pone.0210498 (PMC6353127; doi:10.1371/journal.pone.0210498)
Supplement: S1 Table — (DOCX) [file pone.0210498.s001.docx]

|  | Without statin | With statin | Univariable cox regression | | Multivariable cox regression* | |
| --- | --- | --- | --- | --- | --- | --- |
|  | (N=742) | (N=784) | HR (95% CI) | p Value | HR (95% CI) | p Value |
| Cardiac arrest or Myocardial infarction | 24 (3.2) | 34 (4.3) | 1.39 (0.79-2.45) | 0.256 | 1.18 (0.68-2.06) | 0.559 |
| Cardiac arrest | 9 (1.2) | 16 (2.0) | 1.78 (0.75-4.21) | 0.191 | 1.67 (0.67-4.15) | 0.268 |
| Myocardial infarction | 19 (2.6) | 22 (2.8) | 1.13 (0.56-2.29) | 0.738 | 0.96 (0.48-1.91) | 0.897 |

CI, confidence interval; HR, hazard ratio. Values are n (%).

*Adjusted for age, gender, year, aspirin, calcium-channel blocker, nitrate, nicorandil, trimetazidine, ACE inhibitor and angiotensin receptor blocker.
